# The role of mindfulness and attachment security in facilitating resilience

**DOI:** 10.1186/s40359-022-00772-1

**Published:** 2022-03-16

**Authors:** Fan Yang, Takashi Oka

**Affiliations:** Department of Psychology, College of Humanities and Sciences, Nihon University, 3-chōme-25-40, Sakurajōsui, Setagaya City, Tokyo 156-8550 Japan

**Keywords:** Mindfulness, Attachment, Resilience, Mediating effects

## Abstract

**Background:**

In recent years, there has been growing interest in exploring ways to facilitate positive psychological dispositions, including resilience. The goal of the present study was to explore the possibility that trait mindfulness facilitates attachment security and thus enhances resilience.

**Methods:**

We conducted two studies based on cross-sectional surveys. In Study 1, data of 207 students studying in Japan was collected. In Study 2, we used a different sample of 203 participants and different measurements to replicate the findings of Study 1.

**Results:**

The results of Study 1 revealed that mindfulness positively predicted resilience, while attachment anxiety and avoidance were mediators between mindfulness and resilience. The results of Study 2 showed that mindfulness positively predicted resilience, and the mediating effect of attachment avoidance was significant, but the mediating effect of attachment anxiety was not significant.

**Conclusions:**

It is possible to facilitate attachment security through cultivating trait mindfulness, and in this way, resilience could be enhanced. The effect of different components of mindfulness on attachment and resilience requires further studies.

## Background

With the development of positive psychology [1], there is growing attention to enhancing human positive psychological traits, such as resilience [2]. Though studies have found that mindfulness is a promising variation to facilitate resilience [3], the underlying mechanisms remain unclear. From the information processing theory of mindfulness [4], mindful individuals may decrease attachment insecurity through increasing conscious awareness of automatic responses to threatening cues of intimate relationships. Therefore, the goal of the present study was to explore the possibility that trait mindfulness is associated with resilience through attachment security, which is indicated by low attachment avoidance and attachment anxiety.

Resilience can be defined as the capacity to maintain or recover high well-being when faced with adverse circumstances [5], and Oshio et al. [6] emphasize the mental recovery function of resilience, including three factors: emotion regulation, novelty seeking and positive future orientation. Resilience is not genetically determined [7], which means it is possible to promote individual resilience by intervention. Previous studies have unraveled the positive psychological outcomes of resilience, such as higher self-compassion [8], higher levels of happiness, and life satisfaction [9, 10]. Especially, resilience has been proved to be a protective factor of psychological health during the crisis of COVID-19 across countries [11–13]. Therefore, it is meaningful to explore methods of facilitating resilience.

There is evidence that those who tend to pay attention to their moment-to-moment experience with a non-judgment attitude have a higher level of resilience [14]. Mindfulness could be regarded as enhanced attention to, and awareness of current experience or present reality [15]. Baer et al. [16] proposed a five-facet mindfulness model, including description, observation, nonreactivity, nonjudgement, and acting with awareness. Furthermore, there is evidence indicates that trait mindfulness could be enhanced through training repeatedly [17]. Existing literature shows that mindfulness training could promote resilience [18–20], while trait mindfulness is a predictor of resilience of different samples, such as homeless people [21], salespeople [22] and nursing students [23]. However, the underlying mechanism between trait mindfulness and resilience remains unclear.

Attachment refers to the affectional bond formed between an infant and caregivers during the early years of life [24]. Moreover, attachment also has an influence on adulthood [25]. Adult attachment can be divided into two dimensions of attachment insecurity: attachment avoidance and attachment anxiety [26]. Attachment avoidance refers to the extent to which a person distrusts intimate partners’ good intention and try to ignore their attachment needs of themselves to maintain their independence, while attachment anxiety refers to the degree to which a person worries about being abandoned by their intimate partner. People who score highly on either of these two dimensions are regarded as being insecurely attached.

As forementioned, mindfulness is associated with resilience [14, 18–23] and attachment security is significantly correlated with both resilience and mindfulness [27–30]. From the information processing theory of mindfulness [4], mindful individuals may decrease attachment insecurity through increasing conscious awareness of automatic responses to threatening cues of intimate relationships. Shapiro et al. [31] also pointed out that reperceiving is the meta mechanism of mindfulness, which suggested through mindfulness, one could witness rather than be immersed in their narrative of life story. These refer to that one could shift into the observer of their experience, being able to consciously choose their behaviors, facilitating more adaptive behaviors which are beneficial to resilience. Several studies suggest that attachment could be the mediator between mindfulness and positive psychological outcomes, such as marital satisfaction [32] and stress responses to conflict [33].

Therefore, the goal of the present study was to test the hypothesis that mindfulness is associated with resilience, and attachment avoidance and attachment anxiety were meditators of the relationship between mindfulness and resilience.

### **Hypothesis 1**

Mindfulness is positively associated with resilience.

### **Hypothesis 2**

Attachment avoidance independently mediates the relationship between mindfulness and resilience.

### **Hypothesis 3**

Attachment anxiety independently mediates the relationship between mindfulness and resilience.

Two studies were conducted to test the hypotheses above. In Study 1, data were collected from 207 participants, but it mixed Chinese and Japanese participants. Measurements were burdensome, containing 83 items in total. Moreover, 6 items in the Japanese version of the Adolescent Resilience Scale (ARS) and 10 items in the Chinese version of Experience of the Experiences in Close Relationships Inventory (ECR) were deleted to keep the Japanese version of measurements and the Chinese version of measurements identical in items. These limitations potentially had a negative impact on results. To deal with these limitations, Study 2 was conducted with Japanese participants only, and shorter measurements.

## Study 1

### Methods

#### Participants

Study 1 was a cross-sectional survey. Two hundred and thirty-five students studying in Tokyo took part in Study 1. However, only 207 students correctly answered the attention check test, being regarded as valid data. Among the participants, 51.7% were Japanese, 56% were females (*M*_age_ = 21.52 years, *SD* = 2.85 years), including 107 Japanese students studying in local Universities (freshmen to seniors; 75.7% females, *M*_age_ = 19.97 years, *SD* = 1.34) and 100 Chinese international students studying in Japan (from Japanese language schools and Universities; 52% females, *M*_age_ = 23.17 years, *SD* = 3.10). Because of the coronavirus, we took advantage of online survey websites to deliver measurements and to collect data, Google Form for Japanese participants, and Wen Juan Xing for Chinese participants. These two websites are similar in appearance, though subtly different in colors. We send the Japanese version of measurements to Japanese participants, and the Chinese version of measurements to Chinese participants, because it was convenient for participants to understand their mother language, which could lead to more trustable results. Considering that mindfulness meditation experience may have an impact on effect sizes of the relationship between mindfulness and attachment [34], the experience of mindfulness and meditation training was also asked and was controlled in regression analysis and mediation analysis. According to the self-report, 28.5% of participants have experience with mindfulness training.

#### Measurements

##### Demographic variables

Gender, age, nationality, and the experience of mindfulness training were collected as the demographic variables.

##### Attachment

For Japanese participants, the Japanese version of the Experiences in Close Relationships Inventory (ECR) developed by Nakao and Kato [35] was used to measure attachment anxiety and attachment avoidance of adult attachment. According to ECR, there are 26 items in total, with 9 items for attachment anxiety and 17 items for attachment avoidance. For Chinese participants, the Chinese version of the Experiences in Close Relationships Inventory (ECR) developed by Li and Kato [36] was utilized to measure attachment anxiety and attachment avoidance of adult attachment. To keep the same with the Japanese version, 10 items only existed in the Chinese version were deleted. In both the Japanese version and the Chinese version, each statement used a 7-point Likert-type scale.

##### Mindfulness

We used the Japanese version of the Five Facet Mindfulness Questionnaire (FFMQ) to measure the level of dispositional mindfulness, which was translated by Sugiura et al. [37]. The number of items is 39, and it includes 5 factors: observation, description, non-judgment, non-reaction, and act with awareness. Deng et al. [38] Chinese version of FFMQ was used to measure the level of dispositional mindfulness of Chinese participants. The number of items is 39. In both the Japanese version and the Chinese version, each statement used a 5-point Likert-type scale.

##### Resilience

We used Adolescent Resilience Scale to measure the resilience (ARS) of Japanese participants, which was developed by Oshio et al. [6]. The Chinese version of the Adolescent Resilience Scale was used for Chinese participants, and it was developed by Matsuda et al. [39]. In the Chinese version, there are 15 items on this scale, and it includes three factors: Positive Future Orientation (PFO), Novelty Seeking (NS), Emotional Regulation (ER). To keep the Japanese version the same as the Chinese version, we deleted six items that only exist in the Japanese version. In both the Chinese version and the Japanese version, each statement used a 5-point Likert-type scale.

##### Attention check test

Because the attitude and attention of participants could change during the period of filling the measurement [40], we set three check questions to ensure the validation of collected data and to recognize the random answer of participants. The check tests were respectively ‘Please choose 3’, ‘1 + 4 = ?’, ‘Please choose 2’.

### Results

Data analysis was conducted by using IBM SPSS Statistics Version 21 and the PROCESS Macro for SPSS Version 3.5 [41].

#### Descriptive statistics

Because all data in Study 1 were collected through self-report measurements, we conducted Harman’s single-factor test to examine the common method bias before the data analysis [52]. All subscales of mindfulness, resilience, and attachment were subjected to exploratory analysis, and the unrotated factor solution was examined to determine the number of factors that are necessary to account for the overall variance. This procedure suggested there was no single factor that accounted for the majority of the covariance among the variables (Facor1 accounted for 19.451% of the covariance).

Table 1 reports the Cronbach *α*s and descriptive statistics for the variables used in Study 1, and Tables 2 and 3 reports the bivariate relationships for variables used in Study 1. The results showed that there were significant correlations between attachment anxiety and mindfulness (*r* = − .36, *p* < .001), attachment avoidance and mindfulness (*r* = − .23, *p* < .01), attachment anxiety and resilience (*r* = − .23, *p* < .01), attachment avoidance and resilience (*r* = − .22, *p* < .01), and mindfulness and resilience (*r* = .21, *p* < .01).

**Table 1:** Descriptive statistics for variables used in the Study 1

	Total (*N* = *207*)			Japanese (*N* = *107*)			Chinese (*N* = *100*)		
	*M*	*SD*	*α*	*M*	*SD*	*α*	*M*	*SD*	*α*
Attachment	89.71	21.42	.87	93.71	21.79	.89	85.43	20.27	.84
Anxiety	33.83	11.30	.87	35.34	10.85	.88	32.21	11.61	.86
Avoidance	55.88	17.33	.90	58.37	17.19	.91	53.22	17.16	.89
Mindfulness	114.71	15.96	.80	112.36	16.68	.83	117.22	14.82	.76
Observation	24.25	6.07	.76	23.12	5.84	.75	25.44	6.11	.75
Description	24.27	6.44	.85	23.22	5.99	.83	25.39	6.75	.86
Nonjudgement	22.21	7.18	.86	23.05	7.97	.91	21.31	6.13	.77
Nonreaction	20.31	4.27	.58	19.64	4.51	.65	21.03	3.90	.48
AWA	23.68	6.50	.84	23.33	6.38	.85	24.05	6.64	.86
Resilience	48.11	10.88	.85	44.86	10.26	.85	51.58	10.49	.82
PF0	19.80	6.32	.90	17.79	6.06	.88	21.95	5.88	.89
NS	14.40	4.46	.91	13.19	4.35	.88	15.69	4.22	.93
ER	13.91	4.56	.75	13.89	4.05	.85	13.94	5.06	.60

Anxiety = Attachment Anxiety, Avoidance = Attachment Avoidance, AWA = Acting with Awareness, PFO = Positive Future Orientation, NS = Novelty Seeking, ER = Emotion Regulation

**Table 2:** Correlations among the main variables in Study 1 (N = 207)

	1	2	3	4
1. Attachment insecurity	1			
2. Anxiety	.59***	1		
3. Avoidance	.85***	.08	1	
4. Mindfulness	− .37***	− .36***	− .23**	1
5. Resilience	− .30***	− .23**	− .22**	.21**

Anxiety = Attachment Anxiety, Avoidance = Attachment Avoidance

**p < .01; *** *p* < .001 (2-tailed)

**Table 3:** Correlations among the subscales of variables in Study 1 (N = 207)

	1	2	3	4	5	6	7	8	9	10
1. Attachment	1									
2. Anxiety	.59***	1								
3. Avoidance	.85***	.08	1							
4. Observation	.07	− .01	.09	1						
5. Description	− .24**	− .16*	− .19**	.13	1					
6. Nonjudgement	− .32***	− .35***	− .17*	− .32***	.15*	1				
7. Nonreaction	− .14*	− .11	− .10	.24**	.23**	.05	1			
8. Act with awareness	.30***	− .27***	− .20**	− .08	.25***	.29***	− .04	1		
9. PFO	− .30***	− .23**	− .22**	− .02	.15*	.13	.06	.12	1	
10. NS	− .04	− .03	− .04	.08	.01	− .15*	− .11	− .04	.49***	1
11. ER	− .25***	− .19**	− .19**	− .18*	.12	.31***	.13	.44***	.18*	.01

Anxiety = Attachment Anxiety, Avoidance = Attachment Avoidance. PFO = Positive Future Orientation, NS = Novelty Seeking, ER = Emotion Regulation

**p < .01; *** *p* < .001 (2-tailed)

In addition, the Cronbach *α* of the non-reaction subscale of FFMQ was .58, .65, and .48, and the Cronbach *α* of the emotion regulation subscale of Chinese version ARS was .60.

#### Conditional process model

We conducted the regression model with mindfulness as the independent variable, attachment anxiety and attachment avoidance as mediating variables, resilience as the dependent variable, and nation and experience of mindfulness training as control variables (Table 4) to test the conditional process models [42]. According to the results, variables put together accounted for 16.2% of the total variance of resilience.

**Table 4:** The regression models of variables in Study 1 (N = 207)

Dependent variable	Independent variable	*R*^2^	*F*	*β*	*SE*	*t*
Anxiety	Mindfulness	.143	11.326***	− .258	.048	− 8.760***
	Experience			− 1.771	1.665	− 1.063
	Nation			2.028	1.486	1.364
Avoidance	Mindfulness	.065	4.668**	− .228	.076	− 2.992**
	Experience			− .353	2.667	− .132
	Nation			4.078	2.381	1.713
Resilience	Mindfulness	.163	7.811***	.056	.050	1.134
	Anxiety			− .145	.067	− 2.156*
	Avoidance			− .095	.042	− 2.262*
	Experience			.231	1.597	.145
	Nation			− 5.526	1.439	− 3.841***

anxiety = attachment anxiety, avoidance = attachment avoidance, experience = the experience of mindfulness training

*p < .05; **p < .01; ***p < .001 (2-tailed)

We used 5000 bootstrap samples to test the mediating effect of attachment anxiety and attachment avoidance in relation to mindfulness and resilience (Table 5). The result showed that both the mediating effects of attachment anxiety and attachment avoidance were significant, 95% bootstrap confidence interval of attachment anxiety was (.003, .080), Bootstrap *SE* = .020, and 95% bootstrap confidence interval of attachment avoidance was (.004, .048), Bootstrap *SE* = .012, and the relative mediating effect was 32.174% and 19.130% respectively. The direct effect was not significant, and the 95% bootstrap confidence interval was (− .042, .154), Bootstrap *SE* = .050.

**Table 5:** The bootstrap mediation analysis of mindfulness (independent variable), attachment anxiety and attachment avoidance (meditators) and resilience (dependent variable) in Study 1 (N = 207)

	Effect value	Bootstrap *SE*	Bootstrap 95%*CI*		Relative mediating effect (%)
			*LL*	*UL*	
Total effect	.115	.046	.024	.207	
Direct effect	.056	.050	− .042	.154	
M_1_	.037	.020	.003	.080	32.174
M_2_	.022	.012	.004	.048	19.130
(C_1_)	.016	.023	− .027	.060	

All variables were standardized. M_1_ = Attachment Anxiety, M_2_ = Attachment Avoidance. C_1_ = Z (attachment anxiety)-Z (attachment avoidance). Relative mediating effect = (indirect effect)/(total effect)

### Discussion

#### Common method bias and correlations

No problematic common method bias was found in Study 1, and all measurements in Study 1 except the nonreaction subscale of in Japanese and Chinese version FFMQ, and emotion regulation subscale in Chinese version ARS had appropriate reliability.

Mindfulness correlated with attachment anxiety, attachment avoidance, and resilience. These findings are consistent with previous research [23, 43]. And we also found that resilience correlated with attachment anxiety and attachment avoidance.

#### Test of hypotheses

Bootstrap mediating effect analysis showed that mindfulness was positively associated with resilience (hypothesis 1), and this relationship was mediated by both attachment avoidance (hypothesis 2) and attachment anxiety (hypothesis 3). These results approved the idea that attachment facilitates a resilient mind [44], suggesting that mindful individuals tend to be more securely attached, that is, lower scores in both attachment avoidance and attachment anxiety, which leads to a better ability to recover in the face of suffering. Align with the idea of information process theory [4], the tendency to take a mindful decentered perspective may be associated with increasing conscious awareness of automatic responses to interpersonal information that could trigger the hyperactivation of attachment anxiety and deactivation of attachment avoidance. And according to the model proposed by Shapiro et al. [31], mindful individuals could keep an intimate distance from their thoughts and feelings without believing in them through the conscious awareness of automatic responses. This conscious awareness of automatic responses would make it possible to revise their internal working model, which may further facilitate resilience.

Some limitations exist in Study 1. First, as mentioned before, the Cronbach *α* of the nonreaction subscale of in Japanese and Chinese version FFMQ, and the emotion regulation subscale in Chinese version ARS were relatively low, therefore one may cast doubts on whether the data we measured was reliable. This may be partly caused by the items we deleted. Second, we only used cross-section data in Study 1, which cannot reveal the causal relationship. Third, Japanese participants and Chinese participants were mixed for the reason that during the coronavirus period, it was hard to collect enough data of a specific sample, though we send the mother language version of the measurements to participants, it could still be a confounding variable.

## Study 2

Considering the limitation mentioned in Study 1, Study 2 was conducted to examine the findings of Study 1 with two changes. First, we collected a different Japanese-only sample. Second, we utilized shorter Japanese version measurements.

### Methods

#### Participants

Study 2 was also a cross-sectional survey. Two hundred and eighteen participants were recruited in Tokyo, Japan. Among 218 participants, 203 participants (74 males, 125 females, and 4 others; *M*_*age*_ = 20.72 years, *SD* = 4.78) answered the attention check test correctly, being regarded as valid data. The University Ethical Committee approved this study.

#### Measurements

##### Demographic variables

Gender and age were collected as the demographic variables.

##### Attachment

The nine-item Japanese version of Experience of Close Relationship-Relationship Structure (ECR-RS) [45] was utilized to measure attachment anxiety and attachment avoidance. Participants answer the items on a 7-point scale (1 = not at all, 7 = completely true). Mean scores were calculated as attachment anxiety score and attachment avoidance score.

##### Mindfulness

The fifteen-item Japanese version of the Mindful Attention Awareness Scale (MAAS) was utilized to measure mindfulness, which was developed by Fujino et al. [46]. Participants answer the items on a 6-point scale (6 = not at all, 1 = almost always). The mean score of every item was calculated as a mindfulness score.

##### Resilience

Twenty-five-item Scale for Resilience Scale for Students developed by Saito and Okayasu [47] were utilized to measure resilience. Participants answer the items on a four-point scale (1 = not at all, 4 = completely true). Mean scores were calculated as resilience scores.

##### Attention check test

Because the attitude and attention of participants could change during the period of filling the measurement [40], ‘Please choose 3’, and ‘Please choose 2’ were asked as attention tests. Participants whose answers were wrong would be regarded as invalid data and thus got deleted.

### Results

Data analysis was conducted by using IBM SPSS Statistics Version 21 and the PROCESS Macro for SPSS Version 3.5 [41], the same as Study 1.

#### Descriptive statistics

Considering all data in study 2 were collected through self-report measurements like in Study 1, we conducted Harman’s single-factor test to examine the common method bias before the data analysis [52]. Measurements of mindfulness, resilience, and attachment were subjected to exploratory analysis, and the unrotated factor solution was examined to determine the number of factors that are necessary to account for the overall variance. This procedure suggested there was no single factor that accounted for the majority of the covariance among the variables (Facor1 accounted for 18.909% of the covariance).

Table 6 reports the Cronbach α and descriptive statistics for the variables used in Study 2, and Tables 7 and 8 report the bivariate relationships for variables used in Study 2. The results showed that there were significant correlations between mindfulness and attachment anxiety (*r* = − .200, *p* < .01), mindfulness and attachment avoidance (*r* = − .245, *p* < .001), attachment anxiety and resilience (*r* = − .269, *p* < .001), attachment avoidance and resilience (*r* = − .521, *p* < .001), and mindfulness and resilience (*r* = .268, *p* < .001). In addition, the Cronbach *α* of every measurement ranged from .791 to .913.

**Table 6:** Descriptive statistics for variables used in the Study 2 (N = 203)

	*M*	*SD*	*α*
Mindfulness	3.661	.733	.791
Attachment	2.957	1.153	.850
Anxiety	3.346	1.794	.913
Avoidance	2.762	1.243	.877
Resilience	2.757	.400	.825

Anxiety = Attachment Anxiety, Avoidance = Attachment Avoidance

**Table 7:** Correlations among the main variables in Study 2 (N = 203)

	1	2	3	4	5
1. Mindfulness	1				
2. Attachment	− .280***	1			
3. Anxiety	− .200**	.725***	1		
4. Avoidance	− .245***	.868***	.288***	1	
5. Resilience	.268***	− .514***	− .269***	− .521***	1

Anxiety = Attachment Anxiety, Avoidance = Attachment Avoidance

*p < .05; **p < .01; ***p < .001 (2-tailed)

**Table 8:** The regression models of variables in Study 2 (N = 203)

Dependent variable	Independent variable	*R*^2^	*F*	*β*	*SE*	*t*
Anxiety	Mindfulness	.043	3.000	− .483	.170	− 2.838**
	Gender			.107	.241	.443
	Age			− .018	.026	− .702
Avoidance	Mindfulness	.061	4.279	4.412	.117	− 3.565***
	Gender			− .029	.166	− .176
	Age			− .004	.018	− .213
Resilience	Mindfulness	.306	17.405	.072	.034	2.135*
	Anxiety			− .024	.014	− 1.723
	Avoidance			− .146	.020	− 7.222***
	Gender			− .038	.046	− .820
	Age			.002	.005	.445

Anxiety = Attachment Anxiety, Avoidance = Attachment Avoidance

*p < .05, **p < .01, ***p < .001 (2-tailed)

#### Conditional process model

We conducted the regression model with mindfulness as the independent variable, attachment anxiety and attachment avoidance as mediating variables, resilience as the dependent variable, and gender and age as control variables (Table 4) to test the conditional process models [42]. According to the results, variables put together accounted for 16.2% of the total variance of resilience.

Five thousand bootstrap samples were utilized to test the mediating effect of attachment anxiety and attachment avoidance in the relationship between mindfulness and resilience (Table 9). The result showed that the mediating effect of attachment avoidance was significant, 95% bootstrap confidence interval of attachment avoidance was (.024, .105), Bootstrap *SE* = .020. However, the mediating effect of attachment anxiety was not significant, and the 95% bootstrap confidence interval of attachment anxiety was (− .004, .032), Bootstrap *SE* = .012. The relative mediating effect of attachment avoidance was 42.361%. The direct effect was significant, and the 95% bootstrap confidence interval was (.006, .138), Bootstrap SE = .034.

**Table 9:** The bootstrap mediation analysis of mindfulness (independent variable), attachment anxiety and attachment avoidance (meditators) and resilience (dependent variable) in Study 2 (N = 203)

	Effect value	Bootstrap *SE*	Bootstrap 95%*CI*		Relative mediating effect (%)
			*LL*	*UL*	
Total effect	.144	.037	.071	.217	
Direct effect	.072	.034	.006	.138	
M_1_	.012	.010	− .004	.032	8.333
M_2_	.061	.020	.024	.105	42.361
(C_1_)	− .049	.023	− .099	− .007	

All variables were standardized. M_1_ = Attachment Anxiety, M_2_ = Attachment Avoidance. C_1_ = Z (attachment anxiety)-Z (attachment avoidance). Relative mediating effect = (indirect effect)/(total effect)

### Discussion

The main aim of Study 2 was to replicate the findings of Study 1 through a different Japanese-only sample and different shorter measurements. For this purpose, data of 203 Japanese participants were collected and analyzed. We successfully replicated the findings that mindfulness was positively associated with resilience (hypothesis 1), and attachment avoidance mediated the relationship between mindfulness and resilience (hypothesis 2). However, we failed to replicate the finding that attachment anxiety could mediate the relationship between mindfulness and resilience.

#### Common method bias and correlations

There was no problematic common method bias found in Study 2, and all measurements in Study were of good reliability.

Mindfulness correlated with attachment anxiety, attachment avoidance, and resilience. And we also found that resilience correlated with attachment avoidance and attachment anxiety. These findings are not only consistent with previous research [23, 43], but also replicated the findings of Study 1.

#### Test of hypotheses

As mentioned above, Study 2 once again indicated that mindfulness is positively associated with resilience (hypothesis 1), and the mediating role of attachment avoidance between mindfulness and resilience was significant (hypothesis 2). However, the mediating role of attachment anxiety between mindfulness and resilience was not significant, that is, hypothesis 2 was not supported by Study 2. The reason might partly be that the measurements utilized in Study 2 were different from measurements in Study 1, especially the measurement of mindfulness. According to Shapiro et al. [31], mindfulness consists of three elements: attention, intention, and attitude. In Study 1, we used the FFMQ, which is a five-facet model of mindfulness, including description, observation, nonreactivity, nonjudgement, and acting with awareness. However, in Study 2, we used the MAAS, which is a one-factor structure and does not measure the nuanced elements of mindfulness. According to the attachment theory, anxiously attached individuals tend to be highly aware of cues and information indicating a potential threat, while avoidantly attached individuals tend to ignore attachment needs so that they could keep their internal working models not activated [48]. Therefore, anxiously attached individuals tend to be high in mindful attention, but their attitude may not be non-judgmental, which was emphasized by the FFMQ and not measured by the MAAS. On the other hand, avoidantly attached individuals are likely to avoid paying attention to their attachment needs of themselves, which were both emphasized by FFMQ and MAAS, and thus Study 2 replicated the findings of Study 1.

## General discussion

Since the end of 2019, the Corona Virus Disease 2019 (COVID-19) had a severe impact on the human lifestyle, and it could be important to recognize factors associated with resilience to better cope with changes and challenges. Two cross-sectional studies were conducted to test the possibility that mindfulness facilitates resilience and test the underlying mechanism. Consistent with previous research, we found that mindfulness was positively associated with resilience [14, 18–23], both attachment avoidance and attachment anxiety were negatively associated to mindfulness and resilience in the correlation analysis [27–30]. Furthermore, attachment avoidance could be the mediator of the relationship between mindfulness and resilience. The mediating effect of attachment anxiety was found in Study 1, but not Study 2, and it may partly be due to the differences between FFMQ and MAAS. It requires further studies on the effect of different components of mindfulness on attachment.

In general, our results partly supported the information process model of mindfulness [4], suggesting that mindful individuals could notice their automatic response patterns like the internal working models of attachment, and then make a pause to choose what to do consciously, which lead to more adaptive behavior and thus facilitate resilience. Indeed, previous studies found that mindfulness is associated with adaptive affective response [49], adaptive factors [50] and adaptive development [51], and attachment is likely to be the mediator between the relationship of mindfulness and positive psychological outcomes [32, 33]. The findings of the present study suggest that during the intervention, it is effective for college students to practice mindfulness to facilitate resilience. Specifically, mindful attention is important to reduce attachment avoidance, leading to a higher level of resilience. And it is likely to be important to cultivate a non-judgmental attitude and practice various mindful skills for those who score highly in attachment anxiety to facilitate their resilience.

There are several limitations in the present study. First, though there are two studies in the present study, only self-report measurements were used. Therefore, combined methods and different resources of data are necessary for further studies. Second, to figure out the causal relationship, experiments are necessary. Third, because of the influence of COVID-19, it is hard to collect enough data of both Japanese and Chinese participants in the present study, and thus the international comparison of the relationship among mindfulness, attachment, and resilience needs to be tested in the future. Fourth, the measurements utilized for Japanese participants and Chinese participants in Study 1 were different in languages, which may have an impact on the results. Therefore, it would be helpful for future research to compare the Japanese version and Chinese version of ECR, FFMQ, and ARS to examine the cross-cultural equivalence.

## Conclusions

Study 1 and Study 2 investigated the relationship between mindfulness, resilience, and attachment. Although it had several limitations, it extended the research to the factors associated with resilience. The findings indicated that mindfulness was positively associated with resilience, and attachment avoidance could mediate the relationship between mindfulness and resilience, and the role of attachment anxiety between mindfulness and resilience required further research.

## Data Availability

Data used during these two studies are available from the corresponding author on reasonable request.

## Abbreviations

ARS: Adolescent Resilience Scale
ECR: The Experiences in Close Relationships Inventory
FFMQ: The Five Facet Mindfulness Questionnaire
PFO: Positive Future Orientation
NS: Novelty Seeking
ER: Emotional Regulation
ECR-RS: The Experience of Close Relationship-Relationship Structure
MAAS: The Mindful Attention Awareness Scale
